# Mobile Phone Dependence among Undergraduate Students of a Medical College of Eastern Nepal: A Descriptive Cross-sectional Study

**DOI:** 10.31729/jnma.4787

**Published:** 2020-04-30

**Authors:** Kriti Thapa, Sami Lama, Rita Pokharei, Rambha Sigdel, Surya Prasad Rimal

**Affiliations:** 1 Department of Psychiatric Nursing, B.P. Koiraia Institute of Health Sciences, Dharan, Nepal; 2 Department of Community Health Nursing, B.P. Koiraia Institute of Health Sciences, Dharan, Nepal; 3 Department of Obstetrics and Gynecology, B.P. Koirala Institute of Health Sciences, Dharan, Nepal

**Keywords:** *dental students*, *dependence*, *medical students*, *mobile phones*, *nursing students*

## Abstract

**Introduction:**

Mobile phones are becoming increasingly indispensable in daily life of the students which has resulted in mobile phone dependence. The objective of the study was to find the prevalence of mobile phone dependence among undergraduate students of a medical college of Eastern Nepal.

**Methods:**

A descriptive cross-sectional study was conducted from October 2016 to March 2017 on a total of 390 undergraduate students aged between 17 and 25 years using stratified sampling technique. Students using mobile phones for more than one year was included in the study. Students were requested to complete a pretested self-administered questionnaire which comprised their socio-demographic characteristics, pattern of mobile phone usage and mobile phone addiction index developed by Leung.

**Results:**

The prevalence of mobile phone dependence among the undergraduate students was found to be 85 (21.8%). Mobile phone dependence was found to be related with time spend on mobile; calls per day, money spend on recharge per month and years of ownership of mobile phone. There was no difference between males and females with regard to mobile phone dependence.

**Conclusions:**

The present study found that mobile phone dependence was common among the undergraduate medical students. These results suggest the need to develop educational programme to educate the students to use mobile phone meaningfully.

## INTRODUCTION

Mobile phone use has become a significant part of students life.^1^ Usage of mobile phones is not intended for negative purposes and influence; however, the attitude and time channelled toward these devices has enslaved the students, making them addicts.^2^ In the recent times, the concept of behavioural addiction has gained significant attention.^3–5^ Behavioural addiction for mobile phones has been variously termed as mobile phone dependence, mobile phone problematic use, problem cell phone use, mobile phone abuse and nomophobia.^6–9^

Studies show that excessive mobile phones use can be associated with different aspects or problems of mental health such as anxiety, depression,^7,8^ low self-esteem,^4^ internet addiction,^9^ high impulsivity,^10^ loneliness, social isolation^11^ etc. Students do not necessarily realize their level of dependence to their cell phones.^1^

The objective of this study was to find out the prevalence of mobile phone dependence among undergraduate students of a medical university of Eastern Nepal.

## METHODS

A descriptive cross-sectional study was conducted from October 2016 to March 2017 among undergraduate students of a medical college in Eastern Nepal. The study was approved by Institutional Review Committee of the college on 24th July 2016. Written informed consent was taken from each participants and confidentiality was maintained.

Sample size was calculated using the formula;

$$\begin{array}{ll} \text{n} & \text{= }{\text{Z}}^{\text{2}}\times \text{p }\times \text{q}/{\text{e}}^{\text{2}}\\ & \text{= }{\text{(1.96)}}^{\text{2}}\times 0.396\times \text{(1}-\text{0.396)}/{\left(0.05\right)}^{\text{2}}\\ & \text{= }368 \end{array}$$

Where,

n = required sample size
p = prevalence of mobile phone dependence (taken as 39.6 based on the study done by Aggrawal et al. in India)
q = 1-p
e = margin of error, 5%
Z = 1.96 at 95 % CI

Adding the 10% non-response rate, the sample size that was taken is 405.

A total of 405 students (MBBS, BDS and B.Sc. Nursing) were selected randomly using stratified sampling technique and 15 were excluded from the study due to the incompletely filled questionnaire. Students who were using mobile phones for more than one year were included. Students were requested to complete a pre-tested self-administered questionnaire which comprised their socio-demographic characteristics, pattern of mobile phone usage and a standard 17 items Mobile phone addiction index (MPAI) developed by Leung. The students having scores higher than 51 were considered dependent on mobile. The data were compiled and analysed in SPSS version 16 and expressed as frequency and percentages.

## RESULTS

The prevalence of mobile phone dependence among the undergraduate students was found to be 85 (21.8%) (Table 1).

**Table 1:** Mobile phone dependence among the respondents.

Characteristics	Category	Number of respondents n (%)
Mobile phone dependence	Dependent	85 (21.8)
	Non-dependent	305 (78.2)
Mean MPAI score ±SD = 41.3±13.6		

Table 2 depicts the association of mobile phone dependence with selected socio-demographic variables (Table 2). Mobile phone was found to be related to years of ownership of mobile phone.

**Table 2:** Pattern of mobile phone dependence with selected socio demographic variables. (n = 390)

Variables		Mobile phone dependence	
		Not dependence	Dependent
Age (in years)	17-19	33	10
	20-22	184	54
	23-25	88	21
Sex	Male	138	40
	Female	167	45
Owned mobile phone (in years)	1-3	86	11
	4-6	138	42
	>6	81	32
Department	B.sc Nursing	48	13
	MBBS	186	43
	BDS	71	29

Table 3 reveals the association of mobile phone dependence with pattern of mobile phone use. Mobile phone dependence was found to be related with time spend on mobile, calls per day and money spend on recharge per month (Table 3).

**Table 3:** Pattern of mobile phone dependence with pattern of mobile phone use.

Variables		Mobile phone dependence	
		Not dependence	Dependent
**Time spend on mobile (in hours)**	1-2	107	9
	2-4	115	28
	4-6	44	29
	>6	39	19
**Calls per day**	1-5	228	49
	6-10	47	24
	>10	30	12
**SMS per day**	0	91	31
	1-4	37	168
	5-8	20	7
	>8	26	10
**Money spend on recharge per month (in Rs)**	<Rs. 500	175	21
	Rs. 500-1000	111	50
	>Rs. 1000	19	14

Table 4 shows the pattern of mobile phone use among the BDS and B.Sc. Nursing students (Table 4). The time of maximum use of mobile phone by the students was in the evening. The average amount of money spend was Rs. 400. Majority of the students used the mobile phone for internet use.

**Table 4:** Pattern of mobile phone use among the respondents.

Characteristics	Category	BDS, n	B.Sc. Nursing, n	Total n (%)
**Time spent per day**	**1-2 hours**	28	21	49 (30.4)
	**2-4 hours**	37	24	61 (37.9)
	**4-6 hours**	19	12	31 (19.3)
	**>6 hours**	16	4	20 (12.4)
	**Median: 3 (5-2)**			
**Calls per day**	**1-5**	76	59	135 (83.9)
	**6-10**	15	2	17 (10.6)
	**>10**	9	0	9 (5.6)
	**Median: 3 IQR (4.5-2)**			
**SMS per day**	**40**	11	51 (31.6)	
	**48**	47	95 (59)	51 (31.6)
	**4**	2	6 (3.7)	95 (59)
	**7**	2	9 (5.6)	6 (3.7)
	**Median: 1 IQR (2.0-0.0)**			
Money spent on recharge per month	**<Rs. 500**	48	37	
	**Rs. 500-1000**	46	20	85 (52.8)
	**>Rs. 1000**	6	4	66 (41)
	**Median: 400 IQR (725-200)**			
**Time of maximum use**	**Morning**	2	0	2 (1.24)
	**Afternoon**	6	1	8 (4.3)
	**Evening**	44	21	65 (40.3)
	**Night**	65	41	106 (65.8)
**Place/Situation of maximum use**	**Classroom**	4	0	4 (2.4)
	**Library**	6	0	6 (3.7)
	**Eating**	6	2	8 (4.9)
	**Driving**	0	0	0
	**Room**	98	61	159 (98.7)
**Reason for use**	**Calling family members**	86	54	140 (87)
	**Calling friends**	76	46	122 (75.7)
	**Messaging**	46	28	74 (46)
	**Internet use**	92	57	149 (92.5)
	**Playing games**	54	23	77 (47.8)
	**Listening to music**	55	48	103 (63.9)
	**Taking photos/videos**	50	46	96 (59.6)
	**Study purpose**	2	15	17 (10.5)

In this study, majority of the participants, i.e. 99 (61.5%) were in the age group 20-22 with mean age of 21.20±1.6 years. Among the total participants most of them, 83 (51.6%) have owned mobile phones since 4-6 years. Among the total participants 153 (39.2%) Self-rated themselves to be addicted to mobile phones. Table 5 shows the demographic characteristics of the study population (Table 5).

**Table 5:** Socio-Demographic Variables of the respondents.

Characteristics	Category	Number of students n (%)
**Sex**	**Male**	34 (21.1)
	**Female**	127 (78.9)
**Type of family**	**Joint**	22 (13.7)
	**Nuclear**	139 (86.3)
**Family income status**	**Bad**	4 (2.5)
	**Average**	122 (75.8)
	**Good**	35 (21.7)
**Department**	**B.sc Nursing**	61 (37.9)
	**BDS**	100 (62.1)
**Academic year**	1st year	36 (22.4)
	2nd year	35 (21.7)
	3rd year	35 (21.7)
	4th year	37 (23.0)
	5th year	18 (11.2)

## DISCUSSION

Although, smartphone use has been increasing across all sectors, university students have been seen as the largest consumer group of smartphone services.^12^ Despite the many interesting and useful functions that the mobile phone fulfils in modern society, maladaptive use of mobile phones has been identified, and has been linked with psychological dysfunction, health problems and even psychiatric disorders.^13^ Mobile phone dependence can be considered as a new diagnostic entity as it has properties of excessive use, withdrawal, tolerance and negative repercussions.^6^ Among the total participants of the study, 39.2% self-rated themselves to be addicted to mobile phones. This study found that around 1/4th of the students (21.8%) had mobile phone dependence which is similar to study done among undergraduates medical students in Maharashtra, India where the prevalence was 24.65%.^14^

Different studies conducted among undergraduate medical students in India have found a wide variety of prevalence of mobile phone dependence. The reported prevalence are 39.5% in Bangalore,^15^ 39.9% in Delhi,^16^ 42.6% in West Bengal,^17^ 62% in Tamilnadu and Kerala,^18^ 71.39%^19^ and 82.1%^20^ in two studies done in Maharashtra. A study done in China and Malaysia among medical students found the prevalence of mobile phone dependence to be 29.8%10 and 47.7%^21^ respectively. Similarly, the prevalence of excessive mobile phone was 36.7% in a medical university in Iran.^11^

These discrepancies could be due to the different instruments and classification method used. Moreover, the inconsistencies might be due to the differences among participants in different studies. Yet, these all findings have confirmed that mobile phone dependence is present among the students and is wide spread. The wide prevalence rate identified in various studies is an indicator that it is a potential health concern.

Mean score of mobile phone dependence was found to be 41.3±13.6 in this study which is comparable with the findings of the study done by Leung on 402 adolescents in Hongkong where mean score was found to be 39.93±12.74.^22^ A study done in Japan and West Bengal, India found the mean score of mobile phone dependence to be 23.6±9.123 and 26.5±9.124 respectively. The difference in score could be due to the different instruments used.

This study didn't reveal association between age and mobile phone dependence. This is similar to the findings reported by some previous studies.^14,18,20^ In contrast, study by AzraDaei et al.^25^ and Mayakal et al.^19^ found that age was significantly associated with mobile phone dependence. It should be noted, however, participants in the study were aged between 17 and 25. Thus, the younger age group in this study may be a possible explanation for this finding, because previous studies have provided substantial evidence for the effect of age on mobile phone dependence with young individuals being more likely to demonstrate such behaviours. Yet, the association between age and mobile phone dependence is yet to be clarified by future studies using broader age groups.

There was no significant association between sex with mobile phone dependence in this study. This is similar to the results obtained in several studies.^10,14–16,18,20^ Bianchi and Phillips^4^ stated that there is no difference between male and females with regard to mobile phone addiction.

However, some studies^11,17^ have reported that female participants have a higher prevalence of mobile phone dependence than males. In contrast, a study in Iran^25^ found the prevalence of mobile phone dependence to be more in males. Bianchi and Phillips^4^ suggested that females use mobile for social reasons while males for technology and work. There is still a need for further studies to unravel the inconsistent prevalence of mobile phone dependence in males and females.

This study revealed that duration of mobile phone ownership was significantly associated with mobile phone dependence which is consistent with the finding of the study done by Yildirim et al. in Turkey.^26^ This contradicts with a study done by Jain et al. in central India.^14^ The mobile phone dependence is significant with the time spend on mobile phone which is similar to the study done in university students by Jain et al. in central India,^14^ Pathivara et al. in Bangalore^15^ and Cagan et al. in Turkey.^26^ Choliz stated that mobile phone addiction was determined in the individual whose duration of mobile phone use is approximately two hours.^27^ With the introduction of facility to use internet and social networking sites on mobile phones, the duration spent on mobile phones has considerably increased in turn, increasing the mobile phone addiction.^18^

Kuss et al. found that calls per day were a significant predictor for the presence of mobile phone dependence.^28^ This is consistent with the finding of this study. A study by Myakal et al. in Maharashtra, India found that as money spent per month on mobile phone increased, there was increasing trend of mobile phone dependence.^19^ Similarly, study by Choudhury et al. in West Bengal India found that total recharge in mobile is positively associated with dependence.^24^ This is in accordance with the findings of this study.

This study showed no significant association of mobile phone dependence with the academic year which is similar to the study done by Dixit et al. in Indore.^29^ This is contrary to the findings of some studies. Studies done by Domple et al.^20^ and D.R. et al.^18^ found first year as an independent risk factor for mobile dependence whereas a study done by Myakal et al.^19^ and Dasgupta et al.^17^ found that mobile phone dependence was higher in third year medical students.

Limitation of the present study is that the results are dependent on the assumption that the students gave honest responses to the questionnaire, as it was self-administered.

## CONCLUSIONS

The present study found that mobile phone dependence was common among the undergraduate students and is associated with time spent on mobile in a day, calls per day, money spend on recharge per month and years of ownership of mobile phone. There was no difference between males and females with regard to mobile phone dependence. These results suggest the need to develop educational programme to educate the students to use mobile phone meaningfully.
